# “Put your Hands up in the Air”? The interpersonal effects of pride and shame expressions on opponents and teammates

**DOI:** 10.3389/fpsyg.2015.01361

**Published:** 2015-09-08

**Authors:** Philip Furley, Tjerk Moll, Daniel Memmert

**Affiliations:** ^1^Institute of Cognitive and Team/Racket Sport Research, German Sport University CologneCologne, Germany; ^2^Cardiff School of Sport, Cardiff Metropolitan UniversityCardiff, UK

**Keywords:** emotion expression, pride, shame, interpersonal effects, nonverbal behavior, point-light

## Abstract

The aim of the present research was to investigate the interpersonal effects of pride and shame expressions amongst opponents and teammates in a soccer penalty scenario. Across a series of experiments using the point-light method, pride and shame expressions exerted strong effects upon observers' anticipated emotions, associated cognitions, and performance expectations. Using the Implicit Association Test (IAT) in two pilot studies we demonstrated that the created pride and shame point-light stimuli were implicitly associated with status and performance related attributes. In Experiment 1, observing pride expressions caused opponents to anticipate more negative emotions, cognitions, and lower performance expectancies toward their next performance in comparison with neutral expressions. In contrast, pride expressions led teammates to anticipate more positive emotions (i.e., pride and happiness), cognitions, and performance expectations toward their next performance than neutral expressions (Experiments 2–4). The results are discussed within the emotions as social information (*EASI*, Van Kleef, 2009) framework by arguing that the social context has to be taken into account when investigating the interpersonal effects of emotion expressions. In conclusion, the present research highlights the potential interpersonal influence of the nonverbal expressions of pride and shame in soccer penalty shootouts.

## Introduction

Hardly any other sporting event is characterized by such intense emotional displays in close succession as penalty shootouts in soccer. From one moment to the other excessive celebration, not only of players but of whole nations, might be replaced by excessive tears and misery as ultimate success and failure lie very closely together in these situations. Two important emotions in this respect are pride and shame that recently have received increased research attention in the psychological literature. An important question regarding these emotions is whether the expression of these emotions can merely be regarded an outcome as highlighted by previous research (Tracy and Matsumoto, 2008) or whether these emotional expressions also influence competitive (opponents) and cooperative others (team-members) as indicated by a recent study by Moll et al. (2010).

According to Van Kleef (2009) the psychological study of emotions has primarily focused on intrapersonal effects of emotions and neglected the interpersonal effects. Van Kleef proposed the e*motions as social information model (EASI-model)* to better understand how distinct emotions (expressions) may exert interpersonal effects via communicating specific social information. This model originates from a social-functional perspective to emotion (Parkinson, 1996; Keltner and Haidt, 1999; Shariff and Tracy, 2011) suggesting that emotions not only evolved to prepare individuals to respond adaptively to recurring stimuli but are fundamental in communicating critical social information to coordinate social interactions and relationships. Of particular importance for the present research, several theorists have proposed that emotional expressions can both deliberately and unintentionally be used to influence others (Van Kleef et al., 2011, p. 154): “Emotion is not just a feeling. Emotion is for influence.” In the present paper we follow the call of Van Kleef et al. (2011) of exploring the EASI model in the context of sport performance by investigating the interpersonal effects of the post-performance expressions of pride and shame on competitive (opponents) and cooperative others (team-members) in the soccer penalty shootout situation.

When individuals feel emotions they usually express emotions (there are some exceptions to this statement, e.g., anger might be inhibited if it is not appropriate in a given social context), and these emotion expressions can be observed by others. Pride is elicited after living up to a certain social standard—success, whilst shame is elicited after failing to live up to a certain social standard—failure (Tracy and Robins, 2007b; Tracy and Matsumoto, 2008). Evidence suggests that both pride and shame displays can be reliably recognized (see Martens et al., 2012 for a recent review).

Pride has a distinct and universally recognized expression consisting of an expanded and upright posture, the head tilted slightly upward, a small smile, and arms raised above the head with hands in fists or the hands on the hips (Tracy et al., 2009). This pride expression is argued to promote high status for the expresser. By displaying pride after success, individuals signal their success to others, thereby boosting status and acceptance (Tracy and Robins, 2007a). Further, the experience and display of pride has been associated with dominance, control, expertise, and power (Williams and DeSteno, 2009; Birch et al., 2010; Fischer et al., 2011), activated feelings of confidence (Huang et al., 2010), and making one feel good, particularly about oneself (Martens et al., 2012). More direct evidence comes from IAT studies showing that pride expressions were implicitly linked with high status (e.g., Shariff and Tracy, 2009).

The shame expression consists of the head tilted downward, a lowered eye gaze, and a slumped posture (Keltner, 1995; Tracy and Matsumoto, 2008; Tracy et al., 2009). Experiencing shame has been associated with feeling smaller and inferior to others (Tangney, 1993). Despite these negative feelings, displaying shame may benefit expressers by functioning to appease onlookers after a social transgression (Keltner and Buswell, 1997). That is, by showing shame individuals inform others that they are aware of their failure, and take responsibility for it to maintain respect and to avoid rejection (Gilbert, 2007).

Of particular relevance for the present research is the increasing body of evidence demonstrating that emotions do not only affect those who experience and express them, but also those who perceive those expressions shaping their feelings, thoughts, and actions (Elfenbein, 2007; Hareli and Rafaeli, 2008; Van Kleef, 2009). Strikingly, Moll et al. (2010) demonstrated that 80 per cent of soccer players who celebrated a successful penalty by showing pride (in comparison to the ones who did not show pride after a successful penalty) during penalty shootouts in the European and World Championships between 1972 and 2008 ended up winning the shootout. Similarly, a trend was evident indicating that players who showed nonverbal signs that are typical of a shame display (i.e., gazing down) were less likely to win the shootout. The main rationale of the present research is therefore to investigate if this effect might have been caused (or partly caused) by the fact that the pride and shame displays influenced opponents and team-mates as speculated by Moll and colleagues.

The EASI model suggests two specific mechanisms via which pride and shame expressions influence observers: inferential processes and/or affective reactions. Inferential processes describe how an observer of emotional expressions is able to infer certain information about the internal states (e.g., feelings, attitudes, relational orientations) of other people. Observers use this information to better understand the situation and it helps them to decide on an adaptive response. For example, when one is observing a pride display, one may conclude that this individual has achieved something important (inference), and should be treated in accordance with this achievement (e.g., Parkinson, 1996). In addition, the observed expressions can elicit affective reactions within the observer. One type of affective reaction occurs via the process of emotional contagion whereby individuals catch the expresser's emotions through their facial expressions, bodily movements and postures, or vocalizations (Hatfield et al., 1993).

**Figure 1:**
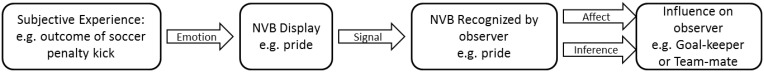
**Hypothesized model based on Van Kleef (2009) on the display of emotional nonverbal expressions in a sports situation**.

Figure 1 displays the combined guiding model for the present research exemplified in a soccer penalty shootout. Depending on the outcome of an important soccer penalty kick, a penalty taker will experience a certain emotion (e.g., pride after a successful attempt and shame after an unsuccessful attempt) which in many cases leads to the nonverbal expression of the respective emotion (Moll et al., 2010). According to evolutionary accounts, the pride and shame expressions signal certain social information which can be reliably recognized by both team-mates and opponents. The EASI model predicts that this influences observer's behavior via the described inferential and affective processes.

Importantly the EASI model further predicts that the relative influence of inferential and affective processes depends on social-contextual factors (Van Kleef, 2009; Van Kleef et al., 2010). Whilst the basic information of distinct emotions generalizes across situations, observers may respond differently to emotional displays depending on the nature of the situation—competitive or cooperative. In competitive situations, the effects of emotion expressions upon observers are driven more by inferential processes and less by affective reactions (Van Kleef et al., 2010). Studies have shown that strategic inferences become more prominent with signs of appeasement leading to less concessions in negotiations (see for a review, Van Kleef et al., 2010). In the case of shame, Tracy and Matsumoto (2008) have argued that displayed shame signals that one places oneself beneath the opponent or aggressor recognizing his/her power and superiority. If so, observers perceiving the display of shame in opponents may infer weakness, which, in turn, may result in opposing thoughts, feelings, and attitudes (e.g., increased confidence, Parkinson, 1996; Van Kleef, 2009). This is not to say that emotional contagion will not occur, but it is less prevalent.

In contrast, when individual's goals are linked in a cooperative manner (e.g., as a team winning the penalty shootout), emotion expressions are more likely to influence observers in a more automatic way through affective reactions (Van Kleef et al., 2010) and less by inferential processes. Indeed, researchers have found that in cooperative situations, observers caught the emotions of the expresser through the process of emotional contagion to, in turn, influence their judgments, decisions, and behaviors (Barsade, 2002; Visser et al., 2013). As alluded to by Moll et al. (2010) displayed pride may induce similar feelings in teammates causing them to experience associated thoughts (e.g., activate feelings of confidence) benefiting subsequent performance. That said, inferential processing may occur as observers can still distill strategic information from the expressions depending on their information processing ability (i.e., low time pressure).

Moll et al. (2010) provided first evidence that post-performance pride expressions had a positive effect on team-mates and a negative effect on opponents when retrospectively analyzing penalty shootouts in soccer. Based on the pattern of results they speculated that pride expressions “(a) caused teammates to feel more confident in taking their own penalty kick; (b) helped to enhance expectancy levels of winning the penalty shootout in teammates; or (c) generally resulted in a more positive approach toward the shootout” (p. 988). In addition, an opponent had over double the chances of missing the next penalty after observing a pride expression by an opponent player in comparison to when a player did not celebrate his success. Although, Moll et al. (2010) reasoned that their findings might be explained via the process of emotional contagion, there is currently no evidence supporting this notion. Further, the fact that pride expressions had a negative impact on opponents seems hard to explain via the proposed emotional contagion mechanism and might be more readily explained via inferential processing (Van Kleef et al., 2010). Hence, we aimed at furthering the understanding of the interpersonal effects of pride and shame expressions on both opponents and team-mates in soccer penalty shootouts We investigated the interpersonal effects of pride and shame expressions in both competitive (Experiment 1) and cooperative social situations (Experiments 2–4). The context of penalty shootouts seems well suited in this endeavor since the emotional expressions in question are displayed frequently (Moll et al., 2010) and easily observable in this situation as the penalty takers are in the center of attention of both opponents and team-mates. Prior to this series of experiments, we used the Implicit Association Test (IAT) to investigate whether pride and shame expressions are implicitly associated with status (Pilot Study 1) and performance (Pilot Study 2) related attributes.

**Figure 2:**
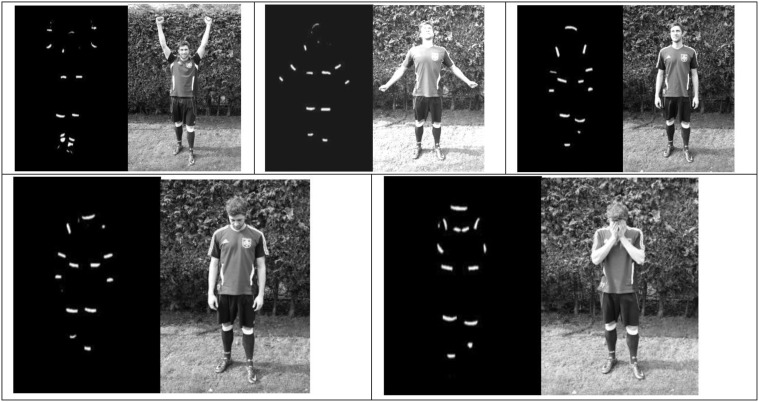
**Single frames of a sample pointlight stimuli used in the study on the left and a picture of the acted behavior on the right**. **Top**: from the left to right: both fists above head, full pride expression, neutral expression; **Bottom**: from left to right: hands in front of face; head down.

We created video footage of penalty takers (Figure 2) using the point-light technique (Johansson, 1973). We chose this method to remove appearance characteristics such as clothing from the display and, more importantly, to examine whether the biological motion information relating to the pride and shame expressions reported in Moll et al. (2010) is sufficient for influencing others. It has been suggested that the accurate inferences drawn from biological motion information may have evolved for fitness reasons in social animals in order to efficiently communicate emotional information with one another (Burgoon, 1996; Blakemore and Decety, 2001; Blake and Shiffrar, 2007; Bente et al., 2010). In support of this, Atkinson et al. (2004) demonstrated that observers could reliably detect emotional states from point-light videos and therefore this approach can be considered a suitable methodology for investigating the interpersonal effects of pride and shame expressions during penalty shootouts. Further, this approach has successfully been employed in previous research investigating nonverbal behaviors (NVB) during the penalty preparation related to dominance and submissiveness (Furley and Dicks, 2012; Furley et al., 2012a) and anxiety (Furley et al., 2012b). If the effects reported by Moll et al. (2010) were indeed due to the interpersonal effects of pride and shame—being automatically related to high and low status (Shariff and Tracy, 2009)—then the scarce biological motion information should be sufficient in influencing soccer players in the penalty shootout situation.

To test this idea we used the created point-light stimulus material in a pilot study to replicate the findings of Shariff and Tracy (2009) who demonstrated that pride expressions serve the distinct evolutionary function of communicating high status, instead of merely positive valence. In addition, we aimed to extend this finding in Pilot study 2 by investigating whether pride and shame expressions are further implicitly linked to performance related attributes. The rationale for using implicit methodologies was to test whether the created point-light stimuli of the pride and shame expressions send automatically perceived social signals that go beyond general positivity and negativity.

## Pilot study 1: Implicit associations between pride and shame expressions and status

Shariff and Tracy (2009) demonstrated that pride expressions serve the distinct evolutionary function of communicating high status, instead of merely positive valence. In this respect, we attempted to use the IAT—which has been successfully used in previous research on NVB and person perception in sports (Furley and Dicks, 2014; Furley and Memmert, 2015)—to replicate their main finding that pride expressions are implicitly linked to high status.

### Methods

#### Participants

Another group of university students (*N* = 21; *M*_age_ = 21.61 years; *SD* = 3.8 years; 10 female), participated in the study. Neither age, nor gender moderated the pattern of results. The study was carried out in full accordance with the Helsinki Declaration of 1975 and written informed consent was obtained from all participants. The study was approved by the local universities ethic committee.

#### Materials, stimuli, and procedure

In order to investigate whether a soccer player displaying pride is implicitly associated with status, we paired the *target-concept* of nonverbal display of pride vs. shame with the *attribute dimension* of high vs. low status, as is standard procedure when using the IAT. For the initial *target concept discrimination*, we selected five images from point-light videos displaying a soccer player displaying pride and five images of a soccer player displaying shame. For the *associated attribute discrimination*, we used the same status related attributes as in Shariff and Tracy (2009): the list contained 5 attributes characteristic (German translation in square parentheses) of a high status individual (commanding [beherrschend]; dominant [dominant]; important [wichtig]; powerful [mächtig]; prestigious [angesehen]) and 5 of a low status individual (humble [demütig]; minor [untergeordnet]; submissive [unterwürfig]; unimportant [unwichtig]; weak [schwach]).

#### Procedure

All participants were seated individually in front of a standard 15 inch notebook computer and provided all their responses via a computer keyboard. Participants were informed that the experiment involved a simple response time test. They were asked to classify images and words as quickly and as accurately as possible and were blind to the actual purpose of the experiment. The procedure used was similar to Greenwald et al. (1998) and consisted of five blocks of trials. The first experimental block (block 3) combined the stimuli from the concept category (proud player/shameful player) with the attribute category (high status/low status), whilst the second experimental block (block 5) reversed this combination. Blocks 1, 2, and 4 were practice blocks for participants to learn the associations between the different stimuli and the respective keys. Depending on the experimental condition, the first experimental block was either congruent concerning our hypothesis (i.e., proud player images paired with high status attributes; and shameful player images paired with low status attributes) and the second experimental block incongruent (i.e., proud player images paired with low status attributes; and shameful player images paired with high status attributes), whereas in the other experimental condition we switched this order to exclude potential order effects. In the congruent condition player images and attributes were randomly presented one by one in the middle of the screen and participants had to press the “q” key for proud player images and good penalty taker attributes, whereas they had to press the “p” key for shameful player images and bad penalty taker attributes. In the incongruent condition participants had to press the “q” key for shameful player images and high status attributes, whereas they had to press the “p” key for proud player images and low status attributes. In addition, the order of blocks 2 and 4 were changed according to the experimental condition to match the attribute categorization of the subsequent experimental blocks 3 and 5.

If the target categories of penalty takers' NVB are differentially associated with the attribute dimension (high vs. low status) as hypothesized, then participants will respond faster to the congruent block in comparison with the incongruent block. After completing the IAT test, participants filled out a questionnaire gathering biographic data.

#### Data analysis

We ran a mixed design ANOVA on the response times of participants with repeated measures on the within subject factors congruency (*congruent* vs. *incongruent*, stimulus material (*player image* vs. *player attributes*), and the between subject factors sequence order (*congruent* before *incongruent* vs. *incongruent* before *congruent*) and type of sport (baseball vs. soccer). We followed up the omnibus ANOVA with a series of dependent t-tests to illuminate the origin of the effects. For the main analysis regarding the comparisons of response time latencies we further report effect size estimates and their precision in form of 95% confidence intervals.

### Results

**Figure 3:**
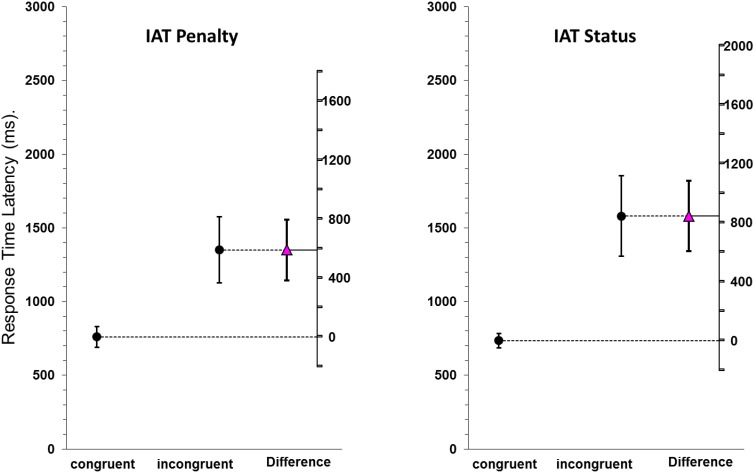
**Mean latency results and 95% confidence intervals for the congruent trials (proud player + good penalty taker; shameful player - bad penalty taker) vs. the incongruent trials (proud player + bad penalty taker; shameful player - good penalty taker) of penalty IAT (left panel) the status IAT (right panel)**. The difference between the group means, with its 95% confidence interval, is shown on a floating difference axis at the right in each panel.

Figure 3 (right panel) displays the mean latencies and the 95% confidence intervals between the congruent block of the IAT (i.e., proud images paired with high status attributes and shameful images paired with low status attributes) and the incongruent block for the status IAT (i.e., proud images paired with low status attributes and shameful images paired with high status attributes). Response time latencies differed substantially between congruent and incongruent trials (M_*difference*_ = 844.67 ms [606.4, 1083.0], *d* = 1.96 [1.15, 2.75]) with participants responding almost a second faster on congruent trials compared to incongruent trials.

The mixed design ANOVA on the response times of participants revealed a significant main effect for congruency [*F*_(1, 19)_ = 127.775, *p* < 0.001, $\eta_{p}^{2}=0.871$], sequence order [*F*_(1, 19)_ = 29.222, *p* < 0.001, $\eta_{p}^{2}=0.606$], and stimulus material [*F*_(1, 19)_ = 9.816, *p* = 0.005, $\eta_{p}^{2}=0.341$]. Further the interaction between congruency and sequence order was significant [*F*_(1, 19)_ = 25.860, *p* < 0.001, $\eta_{p}^{2}=0.576$]. No other interactions reached significance (all *p* > 0.26).

The IAT effect was evident for both penalty taker attributes (congruent: *M* = 811.00 ms; *SD* = 142.00 ms vs. incongruent: *M* = 1616.37 ms; *SD* = 586.03 ms) and player images (congruent: *M* = 659.26 ms; *SD* = 80.39 ms vs. incongruent: *M* = 1543.22 ms; *SD* = 650.80 ms). These results suggest that participants show strong implicit associations between a penalty takers post-performance NVB and attributes related to status. Follow-up dependent *t*-tests revealed significant differences between the congruent and the incongruent conditions for both the player image stimuli (*t*_(20)_ = −6.839, *p* < 0.001, two-tailed, *d* = 1.91 [1.01, 2.70]) and the status attribute stimuli (*t*_(20)_ = −7.401, *p* < 0.001, two-tailed, *d* = 1.89 [1.11, 2.65]).

### Discussion

Results of Pilot Study 1 replicated the findings of Shariff and Tracy (2009) and showed that the pride and shame point-light stimuli were implicitly associated with status-related attributes. Specifically, we found substantially faster reaction times when pride expressions were paired with high status words and shame expressions were paired with low status words compared to when pride expressions were paired with low status words and shame expressions with high status words (Figure 3, right). As participants were equally motivated to respond as quickly as possible on every trial (Shariff and Tracy, 2009), this finding suggests that the stimulus material was differentially associated to status implicitly.

To investigate whether pride and shame expressions might not only be implicitly associated with status related attributes, but further associated with performance related attributes in soccer, we created an additional IAT in Pilot Study 2.

## Pilot study 2: Implicit associations between pride and shame expressions and penalty performance

### Methods

#### Participants

A group of soccer players (*N* = 21; *M*_age_ = 22.0 years; *SD* = 2.5 years; 9 female), who had an average of 13.6 years (*SD* = 4.3) of playing experience, participated in the study. Neither age, gender, nor experience moderated the pattern of results. The study was carried out in full accordance with the Helsinki Declaration of 1975 and written informed consent was obtained from all participants.

#### Materials, stimuli, and procedure

In order to investigate whether a soccer player displaying pride is implicitly associated with attributes characterizing a “good penalty taker,” we paired the *target-concept* of nonverbal display of pride vs. shame with the *attribute dimension* of good vs. bad penalty taker, as is standard procedure when using the IAT. We used the same pride and shame displays as in the previous IAT. For the *associated attribute discrimination*, we initially asked a soccer expert, teaching coaching courses in soccer at the local university, to create a lists consisting of 10 attributes being either associated with a good penalty taker and 10 attributes with a bad penalty taker. In a second step, two different soccer experts (in possession of a high coaching license) rated this list of attributes as being either characteristic of a good penalty taker or of a bad penalty taker on a Likert scale ranging from 1 “very characteristic of a bad penalty taker” to 7 “very characteristic of a good penalty taker.” Following the expert ratings, we produced a list of 5 attributes (German translation in square parentheses) that were rated highest as being characteristic of a good penalty taker (good finishing [abschlussstark]; confident [selbstbewusst]; focused [konzentriert]; composed [gefasst]; assertive [durchsetzungsfähig]) as being rated highest for a bad penalty taker (poor finishing [abschlussschwach]; not confident [nicht selbstbewusst]; distracted [abgelenkt]; on edge [gestresst]; insecure [unsicher]). If the target categories of penalty takers' NVB are differentially associated with the attribute dimension (good vs. bad penalty taker) as hypothesized, then participants will respond faster to the congruent block in comparison with the incongruent block. After completing the IAT test, participants filled out a questionnaire gathering biographic data. Otherwise the procedure was identical to the previous IAT.

### Results

Figure 3 (left panel) displays the mean latencies and the 95% confidence intervals between the congruent block (i.e., proud images paired with positive performance related attributes and shameful images paired with negative performance related attributes) of the IAT and the incongruent block for penalty IAT (i.e., proud images paired with negative performance related attributes and shameful images paired with positive performance related attributes). Response time latencies differed substantially between congruent and incongruent trials (*M*_*difference*_ = 589.88 ms [383.6, 796.2], *d* = 1.62 [0.88, 2.34]) with participants responding over half a second faster on congruent trials compared to incongruent trials.

The mixed design ANOVA on the response times of participants revealed a significant main effect for congruency [*F*_(1, 19)_ = 34.375, *p* < 0.001, $\eta_{p}^{2}=0.644$] and stimulus material [*F*_(1, 19)_ = 28.249, *p* < 0.001, $\eta_{p}^{2}=0.598$]. Further the interaction between congruency and stimulus material was significant [*F*_(1, 19)_ = 7.003, *p* = 0.016, $\eta_{p}^{2}=0.269$]. The main effect for sequence order (*p* = 0.70, $\eta_{p}^{2}=0.008$), nor any of the other interactions reached significance (all *p* > 0.53). The IAT effect was evident for both penalty taker attributes (congruent: *M* = 847.89 ms; *SD* = 190.15 ms vs. incongruent: *M* = 1525.28 ms; *SD* = 599.21 ms) and player images (congruent: *M* = 672.05 ms; *SD* = 131.53 ms vs. incongruent: *M* = 1174.41 ms; *SD* = 435.08 ms).

Follow-up dependent *t*-tests revealed significant differences between the congruent and the incongruent conditions for both the player image stimuli (*t*_(20)_ = −5.623, *p* < 0.001, two-tailed, *d* = 1.56 [0.83, 2.28]) and the player attribute stimuli (*t*_(20)_ = −5.777, *p* < 0.001, two-tailed, *d* = 1.52 [0.8, 2.21]).

### Discussion

The results of Pilot Study 2 suggest that participants further show strong implicit associations between a penalty takers pride and shame displays and attributes related to their penalty taking performance. In tandem with the findings from Pilot Study 1 and the findings from Shariff and Tracy (2009), it therefore seems plausible that pride and shame expressions in a penalty situation have distinct communicative effects by being implicitly related to both status and performance. After validating these distinct implicit associations of pride and shame expressions, we move on to investigating the interpersonal effects of pride and shame expressions on both competitive (Experiment 1) and cooperative observers (Experiments 2–4) in penalty shootouts.

## Experiment 1: The effect of nonverbal pride and shame expressions on opponents

In Experiment 1, we examined the effects of observing post-performance shame and pride expressions among a group of goal-keepers using a within-subject design similar to previous research on nonverbal expressions in sports (Greenlees et al., 2005, 2008; Furley and Dicks, 2012; Furley et al., 2012a,b). Based upon the suggestions of Moll et al. (2010), we hypothesized that pride and shame expressions could be distinguished based on biological motion information from neutral expressions; that pride expressions would lead to more negative anticipated emotions and cognitions compared to a neutral expression; and shame expressions would lead to more positive anticipated emotions and cognitions compared to a neutral expression amongst opposing goal-keepers.

### Methods

#### Participants

Fifteen experienced male goalkeepers (*M*_age_ = 27.1; *SD* = 8.1) took part in the study, who had on average 15 years (*SD* = 7.1) of amateur to semiprofessional playing experience. Neither age nor playing experience significantly moderated the pattern of results. Informed consent was obtained from every participant before commencing the experiment. The study was carried out in accordance with the Helsinki Declaration of 1975.

#### Materials and stimuli

The filming took place in a dark room where almost all ambient light was blocked. The point-light footage was recorded using a Canon HG21 digital video camera mounted on a tripod at a height of 1.85 m, 11 m from a penalty spot resembling the perspective goalkeepers have on the penalty taker. Two halogen spotlights were positioned in front of the camera directed at the actor executing the penalty kick. Four actors were recruited to create the stimulus material. They all received the same instructions on how to execute the penalty kick and how to behave after the kick when being filmed. In Experiment 1 every actor first pretended to execute a penalty kick and then take two steps toward the camera while acting various post-performance expressions detailed below. The actors wore black tight fitting clothes and headwear. The reflective tape was placed on the clothes (Figure 2) following the procedures outlined by Atkinson et al. (2004).

##### Post-performance NVB manipulation

NVBs expressing pride and shame were created based both on the coding system adopted by Tracy and Matsumoto (2008) and on the coding system used by Moll et al. (2010) to make them more representative of the emotional expression during penalty shootouts. Based on Moll et al. (2010) we created six different post-performance NVBs associated with pride, shame, and one neutral NVB expression (cf. Figure 2). The first NVB expression of pride involved the player (i) tilting the head back; (ii) extending both arms above the head with hands in fists; and (iii) expanding the chest (cf. left most image of the top panel of Figure 2). The second one involved the actor (i) tilting the head back, (ii) expanding the chest, (iii) turning the shoulders outward with the hands facing the camera, and (iv) the arms slightly extended from the body (cf. middle image of the top panel of Figure 2). The neutral condition involved the actor neutrally taking two steps toward the goalkeeper after the penalty execution (cf. right most image of the top panel of Figure 2). In the neutral condition we asked participants to (i) adopt a relaxed stance with the feet shoulder-wide apart and the shoulders casually hanging; (ii) neither collapse the limbs inwards nor outwards; (iii) not to deliberately hold the head up and the chin slightly pointed toward the ground. The leftmost image of the bottom panel of Figure 2 shows the first shame expression which simply involved the actor to (i) gaze down with (ii) the shoulders slumped. The rightmost image of the bottom panel of Figure 2 shows the second shame expression that involved a (i) slumped posture and (ii) moving the hands in front of the face to cover it. We implemented two versions of this shame expression one involving gazing down and the other tilting the head back as these were differentiated in Moll et al. (2010). However, as these were literally perceived as identical on all ratings we did not differentiate between these in the data analysis and pooled them as one expression of shame.

##### Stimuli selection

Each actor was filmed in the 6 different emotional expression conditions three times, before one video from each condition was selected by the experimenters that was—except for the experimental manipulation—most similar to one another. Hence, the final experiment contained 24 point-light videos of approximately 4 s length—4 actors in the 6 experimental conditions which we reduced to five in the data analysis as the two shame conditions that involved hiding the face behind the hands while either facing down or up were rated identically and therefore were pooled to one condition.

#### Measures

After every video, participants rated the observed player on several computer-generated 11-point digital semantic differential scales. The measures were partially derived from previous person perception research in sports (cf. Furley et al., 2012a,b), from previous research on pride (Williams and DeSteno, 2008), whereas others were included in an exploratory manner. In order to give their ratings, participants had to move a mouse cursor from the middle of the scale toward either end of the scale and provide their rating by clicking the left mouse button. The E-prime software transformed the ratings into a value (with 3 decimals) between 0 reflecting the left end of the scale and 1 reflecting the right end of the scale.

##### Perception of target player

The first seven measures provided data on the perceived impressions of the observed penalty taker and served as a manipulation check. The dimensions were: (i) not confident–confident; (ii) on edge–composed; (iii) stressed–relaxed; (iv) unhappy–happy; (v) calm–excited; (vi) not ashamed–ashamed; and (vii) not proud–proud.

##### Expected feelings/cognitions items

Participants rated their anticipated feelings/cognitions after viewing the emotion expression on the following items: First, participants rated their anticipated feelings of pride, shame, and happiness toward the next penalty with the following three items: (i) not proud–proud; (ii) ashamed–not ashamed; (iii) unhappy–happy. To assess how stressful participants anticipated feeling toward the next penalty, they rated the following items: (iv) on edge–composed; (v) stressed–relaxed; (vi) excited—calm; and (vii) worried–content. Participants rated their anticipated thoughts toward the next penalty on the following items: (viii) not confident–confident; (ix) not in control–in control; (x) not focused–focused; (xi) uncomfortable–comfortable.

##### Expected quality of next penalty and performance toward shootout

Participants rated their expectancy of the power of the penalty kick along the dimensions very weak—very powerful with low scores reflecting weak penalties. Further they rated the expected accuracy of the penalty kick along the dimensions very inaccurate—very accurate with low scores reflecting inaccurate penalties.

The next three items assessed the extent to which participants expected to: (i) perform to the best of their ability; (ii) to save the next penalty; and (iii) to win the shootout. Participants had to give their ratings along the dimensions not sure at all and very sure.

#### Procedure

E-prime 2.0 professional (Psychological Software Tools, 2007) was used to present the stimuli and collect the judgments on a 17-inch computer screen placed 60 cm away from the subjects. Every participant viewed the 24 experimental videos in a random order. Participants were instructed that they had to assume the role of the opposing goal-keeper in a penalty shootout situation and that point-light video clips would be presented of different penalty takers performing penalty kicks. Subsequently they were informed that they would have to answer questions about the penalty taker, the next penalty in line, and the entire shootout based solely on the penalty footage that was presented to them in the point-light displays. Before commencing the experiment, participants filled out a questionnaire gathering demographic data. Every participant was tested individually. Participants first viewed a point-light video to familiarize themselves with the procedure prior to the 24 experimental clips that were presented in random order. After completing the Experiment, participants were informed about the purpose of the study.

#### Data analysis

We calculated a series of within subject ANOVAs with repeated measures on the within subject independent variable post-performance NVB (fists above head; chest expanded; neutral; head down; and hands in front of face) on the seven perception of target player items, on the eleven feelings/cognitions toward next penalty items, the two expected penalty quality items, and the three expected performance items. Further, we conducted a series of planned contrasts testing the respective pride and shame expressions against the neutral expression for every dependent variable. Where, the assumption of sphericity was violated, the *p*-values were computed using the conservative Greenhouse-Geisser method with corrected degrees of freedom.

### Results

#### Perception of target player and manipulation check

The univariate analysis and descriptive statistics of the seven perception of target player scales that served as a manipulation check are displayed in Table 1. The results revealed that the manipulated post-performance pride and shame expressions were recognized by the observers. Especially the large effect sizes for the proud and shame scales highlight the successful manipulation of the displayed NVB in question. However, it should be noted that the effect sizes for happiness were similarly high. A point we will return to in the general discussion section. Planned contrasts revealed that the fist above head expression significantly differed (except marginally nonsignificant for the calm-excited measure; *p* = 0.055; $\eta_{p}^{2}=0.24$) from the neutral condition on all the dependent measures (all $\eta_{p}^{2}>$ 0.80). Similarly, the chest expanded condition significantly differed from the neutral condition on all measures except marginally not for calm-excited (*p* = 0.063; $\eta_{p}^{2}=0.24$) and not ashamed-ashamed (*p* = 0.083; $\eta_{p}^{2}=0.20$). Target players were rated as more confident, more composed, more relaxed, happier, and as less ashamed when displaying pride as compared to the neutral expression. Both shame expressions differed significantly on all the dependent measures from the neutral condition (all $\eta_{p}^{2}>$ 0.40), except on the calm-excited measure between neutral and head-down (*p* = 0.095; $\eta_{p}^{2}=0.19$). Target players were rated as less confident, less happy, more on edge, more stressed, more excited, and as more ashamed when displaying shame as compared to the neutral expression.

**Table 1:** **Univariate analysis of Experiment 1 (opponent goal-keepers) for the main effects of post-performance NVB on the perception of the target player**.

**Item**	***M*(*SD*) NVB1**	***M*(*SD*) NVB2**	***M*(*SD*) NVB3**	***M*(*SD*) NVB4**	***M*(*SD*) NVB5**	***df* (model, error)**	***F***	**η^2^*p***	***p***
Not confident–confident	0.95(0.06)	0.76(0.07)	0.54(0.08)	0.14(0.08)	0.07(0.08)	4, 56	346.5	0.96	<0.001
On edge–composed	0.93(0.05)	0.81(0.07)	0.74(0.11)	0.53(0.30)	0.17(0.13)	1.9, 25.9	56.4	0.80	<0.001
Stressed–relaxed	0.92(0.07)	0.80(0.08)	0.73(0.10)	0.51(0.30)	0.15(0.12)	1.8, 24.9	57.2	0.80	<0.001
Unhappy–happy	0.97(0.03)	0.76(0.06)	0.49(0.07)	0.12(0.07)	0.06(0.04)	4, 56	624.2	0.98	<0.001
Calm–excited	0.44(0.35)	0.33(0.14)	0.25(0.09)	0.38(0.28)	0.78(0.16)	1.6, 22.7	11.0	0.44	<0.001
Not ashamed–ashamed	0.02(0.02)	0.21(0.08)	0.27(0.18)	0.88(0.11)	0.93(0.07)	1.7, 24.3	307.7	0.96	<0.001
Not proud–proud	0.99(0.02)	0.81(0.06)	0.51(0.06)	0.10(0.08)	0.03(0.02)	2.8, 38.5	922.4	0.99	<0.001

*NVB1, fists above head; NVB2, chest expanded; NVB3, neutral; NVB4, head down; NVB5, hands in front of face*.

*The left pole of the scale (e.g., not confident) = 0.00 and the right side of the pole = 1.00 (e.g., confident)*.

#### Expected feelings/cognitions items

The univariate analysis and descriptive statistics of the eleven anticipated feelings and thoughts toward the next penalty kick scales are displayed in Table 2.

**Table 2:** **Univariate analysis of Experiment 1 for the main effects of post-performance NVB on the expected feelings/cognitions items**.

**Item**	***M*(*SD*) NVB1**	***M*(*SD*) NVB2**	***M*(*SD*) NVB3**	***M*(*SD*) NVB4**	***M*(*SD*) NVB5**	***df* (model, error)**	***F***	**η^2^*p***	***p***
Not proud–proud	0.24(0.14)	0.41(0.17)	0.53(0.14)	0.85(0.09)	0.92(0.05)	1.7, 24.4	136.3	0.90	0.000
Not ashamed–ashamed	0.73(0.12)	0.58(0.17)	0.45(0.15)	0.14(0.09)	0.09(0.07)	1.9, 26.5	94.4	0.87	0.000
Unhappy–happy	0.20(0.10)	0.38(0.07)	0.50(0.10)	0.84(0.10)	0.90(0.06)	1.8, 26.0	175.3	0.93	0.000
On edge–composed	0.24(0.11)	0.38(0.08)	0.50(0.10)	0.85(0.07)	0.88(0.06)	1.8, 25.7	221.2	0.94	0.000
Stressed–relaxed	0.23(0.09)	0.38(0.07)	0.50(0.10)	0.85(0.08)	0.89(0.06)	2.0, 28.2	231.6	0.94	0.000
Calm–excited	0.79(0.12)	0.62(0.16)	0.51(0.13)	0.16(0.10)	0.10(0.08)	1.5, 21.2	135.0	0.90	0.000
Worried–content	0.22(0.06)	0.35(0.07)	0.47(0.07)	0.79(0.11)	0.82(0.11)	1.9, 25.9	174.9	0.93	0.000
Not confident–confident	0.62(0.20)	0.66(0.15)	0.69(0.10)	0.89(0.07)	0.90(0.05)	1.7, 23.1	40.6	0.74	0.000
Not in control–in control	0.66(0.18)	0.68(0.12)	0.67(0.10)	0.84(0.10)	0.88(0.07)	1.4, 19.5	27.9	0.66	0.000
Not focused–focused	0.74(0.13)	0.74(0.12)	0.75(0.10)	0.87(0.09)	0.90(0.06)	1.5, 20.9	31.2	0.69	0.000
Uncomfortable–comfortable	0.23(0.08)	0.38(0.08)	0.49(0.09)	0.80(0.08)	0.84(0.06)	2.2, 30.6	197.1	0.93	0.000

*NVB1, fists above head; NVB2, chest expanded; NVB3, neutral; NVB4, head down; NVB5, hands in front of face*.

*The left pole of the scale (e.g., not proud) = 0.00 and the right side of the pole = 1.00 (e.g., proud)*.

Planned contrasts revealed that the fist above head expression significantly differed from the neutral condition on most of the expected feelings and cognition measures (all $\eta_{p}^{2}>$ 0.82 for the significant measures), except for confidence (*p* = 0.091; $\eta_{p}^{2}=0.19$), focus (*p* = 0.921; $\eta_{p}^{2}=0.01$), and control (*p* = 0.797; $\eta_{p}^{2}=0.01$). A similar pattern was evident for the comparisons between the chest expanded and the neutral condition (all $\eta_{p}^{2}>$ 0.52 for the significant measures), showing significant differences between all measures except for the confidence (*p* = 0.244; $\eta_{p}^{2}=0.10$), focus (*p* = 0.858; $\eta_{p}^{2}=0.01$), and control (*p* = 0.431; $\eta_{p}^{2}=0.05$) measures as for the other pride expression. Opposing goalkeepers expected to feel less proud, more ashamed, more unhappy, more on edge, more stressed, more excited, more worried, and more uncomfortable when observing an opposing penalty taker display pride as compared to a neutral expression. Both shame expressions significantly differed from all the expected feelings and cognitions scales compared to the neutral condition (all $\eta_{p}^{2}>$ 0.76). Opposing goalkeepers expected to feel prouder, less ashamed, happier, more composed, more relaxed, calmer, more content, more confident, more in control, more focused and more comfortable when observing an opposing penalty taker display shame as compared to a neutral expression.

#### Expected quality of next penalty kick and performance toward shootout

The univariate analysis and descriptive statistics of the two expected quality scales and the three confidence scales are displayed in Table 3.

**Table 3:** **Univariate analysis of Experiment 1 for the main effects of post-performance NVB on the anticipated next penalty quality and the expected performance toward shootout**.

**Item**	***M*(*SD*) NVB1**	***M*(*SD*) NVB2**	***M*(*SD*) NVB3**	***M*(*SD*) NVB4**	***M*(*SD*) NVB5**	***df* (model, error)**	***F***	**η^2^*p***	***p***
Inaccurate–accurate	0.87(0.08)	0.72(0.06)	0.57(0.06)	0.25(0.12)	0.21(0.13)	1.3, 18.8	131.5	0.90	0.000
Weak–powerful	0.85(0.10)	0.69(0.06)	0.57(0.06)	0.25(0.13)	0.21(0.12)	1.3, 18.7	111.3	0.88	0.000
Perform to best of ability	0.59(0.18)	0.63(0.15)	0.67(0.13)	0.85(0.08)	0.89(0.06)	1.4, 19.2	52.3	0.79	0.000
Saving penalty	0.54(0.21)	0.61(0.16)	0.66(0.13)	0.86(0.07)	0.88(0.06)	1.5, 20.7	48.7	0.77	0.000
Winning shootout	0.56(0.21)	0.62(0.16)	0.67(0.12)	0.90(0.07)	0.92(0.05)	1.4, 20.1	52.5	0.79	0.000

*NVB1, fists above head; NVB2, chest expanded; NVB3, neutral; NVB4, head down; NVB5, hands in front of face*.

*The left pole of the scale (e.g., inaccurate) = 0.00 and the right side of the pole = 1.00 (e.g., accurate)*.

Planned contrasts revealed that both pride expressions significantly differed from the neutral condition on all of the expected quality of penalty kick and performance measures (all *p* < 0.012; all $\eta_{p}^{2}>$ 0.37). Opposing goalkeepers expected a more accurate penalty kick, a more powerful penalty kick, and to perform worse in the shootout when observing an opposing penalty taker display pride as compared to a neutral expression.

The same was true for the two shame expressions (all *p* < 0.012; all $\eta_{p}^{2}>$ 0.77). Opposing goalkeepers expected to feel prouder, less ashamed, happier, more composed, more relaxed, calmer, more content, more confident, more in control, more focused, and more comfortable when observing an opposing penalty taker display shame as compared to a neutral expression.

#### Control group of outfield players

In order to replicate the pattern of results (i.e., positive emotion expressions have a negative effect and negative expressions a positive effect on opponents) amongst opponent goalkeepers, we additionally tested a group of 20 experienced male outfield players (*M*_age_ = 24.8; *SD* = 6.3) who had on average 17 years (*SD* = 3.0) of amateur to semiprofessional playing experience (using the stimulus material from Experiment 2 which showed the players perspective instead of the goalkeeper perspective). The outfield players were asked to assume the role of the next opponent penalty taker in line and give their ratings toward their next penalty kick. The pattern of results amongst opponent penalty takers was almost identical to opponent goalkeepers. When factoring in the between group independent variable (goalkeepers/players) the Two-Way mixed ANOVA did not reveal any between group main effects on any of the dependent variables (all *p* > 0.3) and showed a very similar pattern of results compared to the goalkeepers, scrutinizing the finding that displayed pride had a negative effect on opponents and displayed shame had a positive effect upon opponents.

### Discussion

The results obtained in Experiment 1 suggest that pride and shame expressions displayed by a player after taking a penalty kick can be recognized—although the results indicate that they might not be distinguishable from happy and unhappy expressions when only having access to biological motion information. More importantly, on the whole, the results revealed that opposing goal-keepers (and outfield players) who observed players displaying pride anticipated to: (i) feel less good in terms of higher levels of shame, lower levels of pride and happiness, (ii) feel more stressed; (iii), less positive cognitions by being less confident, in control, focused, and comfortable; and (iv) lower performance quality and expectations in the shootout compared to when observing players displaying a neutral expression. Opposing results were obtained for those goal-keepers (and outfield players) who observed players displaying shame compared to players displaying a neutral expression. These findings suggest that in a competitive context, pride and shame expressions cause opposing feelings and thoughts in observers.

In Experiment 2, we focused on the effects of pride and shame displays upon cooperative others (teammates).

## Experiment 2: The effect of nonverbal pride and shame expressions on team-mates

In Experiment 2, we investigated the effects of observing nonverbal expressions of pride and shame on team-mates during a soccer penalty shootout as, according to the EASI-model, it depends on the nature of the situation—competitive or cooperative (Van Kleef et al., 2010) how observers respond to these emotion displays. We hypothesized that the expressions of pride and shame would have different interpersonal effects on the observer if the target was a cooperative team-member as opposed to an opponent as in Experiment 1. After observing pride, we predicted that teammates would anticipate experiencing more positive emotions and higher levels of associated cognitions (e.g., confidence, control, performance expectations). After observing displayed shame, we predicted that teammates would anticipate experiencing more negative emotions and lower levels of associated cognitions. Hence, we predicted that pride expressions would differ from neutral expressions and shame expressions would differ from the neutral expressions on the corresponding measures.

### Methods

#### Participants

Sixteen experienced male outfield players took part in the study (*M*_age_ = 23.4; *SD* = 2.2), who had on average 15 years (*SD* = 3.2) of amateur to semiprofessional playing experience. Neither age nor playing experience significantly moderated the pattern of results. Informed consent was obtained from every participant before commencing the experiment. The study was carried out in accordance with the Helsinki Declaration of 1975.

#### Materials and procedure

The materials and procedure in Experiment 2 were identical to Experiment 1, except for the following changes: We created new point-light stimuli resembling the view that team-mates and opponent penalty takers have when viewing the shootout. This time the actors were filmed from behind. After executing the penalty the actors were instructed to turn round and jog toward the camera while displaying the NVBs in question. The experimental manipulation was identical to Experiment 1; Further, participants were told that they had to take over the role of the penalty taker next in line and give their ratings toward their next penalty kick; The only other difference was that one of the outcome expectation scales was changed and participants had to rate how confident they were that they would score the next penalty. Otherwise, everything was identical to Experiment 1.

### Results

#### Perception of target player and manipulation check

The univariate analysis and descriptive statistics of the seven perception of target players scales replicated the findings of Experiment 1 (cf. Table 4). This confirms that both pride and shame are recognized by others, although they might not be distinguishable from happy and unhappy.

**Table 4:** **Univariate analysis of Experiment 2 (own players) for the main effects of post-performance NVB on the perception of the target player**.

**Item**	***M*(*SD*) NVB1**	***M*(*SD*) NVB2**	***M*(*SD*) NVB3**	***M*(*SD*) NVB4**	***M*(*SD*) NVB5**	***df* (model, error)**	***F***	**η^2^*p***	***p***
Not confident–confident	0.78(0.20)	0.67(0.09)	0.66(0.09)	0.38(0.21)	0.31(0.25)	1.6, 23.4	20.0	0.57	0.000
On edge–composed	0.69(0.20)	0.62(0.09)	0.63(0.08)	0.56(0.19)	0.31(0.21)	2.2, 33.3	11.6	0.44	0.000
Stressed–relaxed	0.69(0.19)	0.61(0.09)	0.64(0.08)	0.56(0.17)	0.28(0.21)	2.1, 31.6	13.9	0.48	0.000
Unhappy–happy	0.84(0.16)	0.60(0.14)	0.60(0.09)	0.28(0.21)	0.19(0.16)	1.5, 21.8	41.6	0.74	0.000
Calm–excited	0.54(0.15)	0.39(0.08)	0.38(0.08)	0.43(0.17)	0.70(0.21)	2.0, 30.1	11.9	0.82	0.000
Not ashamed–ashamed	0.17(0.17)	0.36(0.13)	0.36(0.07)	0.72(0.21)	0.80(0.19)	1.6, 23.9	37.7	0.71	0.000
Not proud–proud	0.83(0.17)	0.60(0.16)	0.62(0.11)	0.29(0.20)	0.21(0.16)	1.7, 25.3	35.1	0.70	0.000

*NVB1, fists above head; NVB2, chest expanded; NVB3, neutral; NVB4, head down; NVB5, hands in front of face*.

*The left pole of the scale (e.g., not confident) = 0.00 and the right side of the pole = 1.00 (e.g., confident)*.

Planned contrasts revealed that the fist above head expression significantly differed from the neutral condition on most of the perception of target player measures (all $\eta_{p}^{2}>$ 0.27 for the significant measures), except for on edge-composed (*p* = 0.255; $\eta_{p}^{2}=0.09$) and stressed-relaxed (*p* = 0.328; $\eta_{p}^{2}=0.06$). Target players were rated as more confident, happier, more excited, prouder, and as less ashamed when displaying pride as compared to the neutral expression. The chest expanded pride expression did not differ on any of the perception of target player measures from the neutral condition (all *p* > 0.388; all $\eta_{p}^{2}<$ 0.05). The hands in front of face shame expression significantly differed from the neutral expression on all these measures (all $\eta_{p}^{2}>$ 0.65 for the significant measures), whereas the gaze down expression (all $\eta_{p}^{2}>$ 0.66 for the significant measures) did not differ from the neutral expression on the on edge-composed (*p* = 0.221; $\eta_{p}^{2}=0.10$); the stressed-relaxed (*p* = 0.157; $\eta_{p}^{2}=0.13$), and calm-excited (*p* = 0.308; $\eta_{p}^{2}=0.07$) measures. Collapsing over both shame expressions, target players were rated as less confident, less happy, more on edge, more stressed, more excited, and as more ashamed when displaying shame as compared to the neutral expression.

#### Expected feelings/cognitions items

The univariate analysis and descriptive statistics of the eleven anticipated feelings toward the next penalty kick scales are displayed in Table 5.

**Table 5:** **Univariate analysis of Experiment 2 for the main effects of post-performance NVB on the expected feelings items**.

**Item**	***M*(*SD*) NVB1**	***M*(*SD*) NVB2**	***M*(*SD*) NVB3**	***M*(*SD*) NVB4**	***M*(*SD*) NVB5**	***df* (model, error)**	***F***	**η^2^*p***	***p***
Not proud–proud	0.76(0.17)	0.62(0.12)	0.59(0.11)	0.40(0.17)	0.36(0.22)	1.2, 18.2	16.6	0.53	0.000
Not ashamed–ashamed	0.25(0.15)	0.36(0.08)	0.37(0.07)	0.57(0.20)	0.61(0.25)	1.2, 17.5	13.3	0.47	0.001
Unhappy–happy	0.76(0.14)	0.60(0.09)	0.61(0.07)	0.39(0.19)	0.33(0.21)	1.2, 18.6	19.6	0.57	0.000
On edge–composed	0.72(0.16)	0.60(0.10)	0.59(0.08)	0.37(0.17)	0.33(0.21)	1.3, 19.1	17.3	0.54	0.000
Stressed–relaxed	0.71(0.17)	0.60(0.10)	0.58(0.08)	0.39(0.15)	0.33(0.21)	1.2, 18.3	15.6	0.51	0.001
Calm–excited	0.37(0.20)	0.44(0.12)	0.45(0.11)	0.61(0.14)	0.70(0.19)	1.4, 20.7	10.5	0.41	0.002
Worried–content	0.71(0.17)	0.58(0.11)	0.59(0.10)	0.40(0.14)	0.33(0.17)	1.4, 21.2	17.5	0.54	0.000
Not confident–confident	0.77(0.14)	0.62(0.11)	0.64(0.12)	0.43(0.22)	0.39(0.25)	1.5, 21.9	14.0	0.48	0.000
Not in control–in control	0.78(0.14)	0.64(0.11)	0.66(0.11)	0.47(0.23)	0.45(0.27)	1.2, 17.6	12.4	0.45	0.002
Not focused–focused	0.85(0.12)	0.73(0.14)	0.70(0.17)	0.57(0.29)	0.53(0.33)	1.3, 18.7	11.2	0.43	0.002
Uncomfort.–comfortable	0.63(0.23)	0.59(0.11)	0.55(0.10)	0.38(0.16)	0.35(0.20)	1.3, 18.9	8.5	0.36	0.006

*NVB1, fists above head; NVB2, chest expanded; NVB3, neutral; NVB4, head down; NVB5, hands in front of face*.

*The left pole of the scale (e.g., not proud) = 0.00 and the right side of the pole = 1.00 (e.g., proud)*.

Planned contrasts revealed that the fist above head expression significantly differed from the neutral condition on most of the expected feelings and cognition measures (all $\eta_{p}^{2}>$ 0.23 for the significant measures), except for on calm-excited (*p* = 0.155; $\eta_{p}^{2}=0.16$). Teammates expected to feel prouder, less ashamed, happier, more composed, more relaxed, more content, more confident, more in control, more focused and more comfortable when observing a penalty taker from the own team display pride as compared to a neutral expression. Again, the chest expanded pride expression did not differ on any of the perception of target player measures from the neutral condition (all *p* > 0.166; (all $\eta_{p}^{2}<$ 0.12). The hands in front of face shame expression and the gaze down shame expression significantly differed from the neutral expression on all the expected feelings and cognition measures (all *p* < 0.008; (all $\eta_{p}^{2}>$ 0.31). Teammates expected to feel less proud, more ashamed, more unhappy, more on edge, more stressed, more excited, more worried, less confident, less in control, less focused, and more uncomfortable when observing an opposing penalty taker display shame as compared to a neutral expression.

##### Expected quality of next penalty and confidence toward shootout

The univariate analysis and descriptive statistics of the two expected quality scales and the three expected performance scales are displayed in Table 6.

**Table 6:** **Univariate analysis of Experiment 2 for the main effects of post-performance NVB on the anticipated next penalty quality and the expected performance toward shootout**.

**Item**	***M*(*SD*) NVB1**	***M*(*SD*) NVB2**	***M*(*SD*) NVB3**	***M*(*SD*) NVB4**	***M*(*SD*) NVB5**	***df* (model, error)**	***F***	**η^2^*p***	***p***
Inaccurate–accurate	0.75(0.15)	0.66(0.06)	0.66(0.11)	0.42(0.24)	0.36(0.23)	1.9, 28.1	18.8	0.56	0.000
Weak–powerful	0.70(0.20)	0.65(0.14)	0.62(0.14)	0.51(0.26)	0.44(0.25)	1.3, 19.7	5.25	0.26	0.025
Perform to best of ability	0.74(0.18)	0.68(0.14)	0.63(0.16)	0.47(0.22)	0.45(0.26)	1.2, 18.6	11.8	0.44	0.002
Scoring penalty	0.76(0.16)	0.67(0.12)	0.62(0.14)	0.45(0.21)	0.42(0.25)	1.3, 19.1	15.0	0.50	0.001
Winning shootout	0.76(0.19)	0.66(0.10)	0.62(0.16)	0.43(0.21)	0.38(0.23)	1.6, 24.2	13.1	0.48	0.000

*NVB1, fists above head; NVB2, chest expanded; NVB3, neutral; NVB4, head down; NVB5, hands in front of face*.

*The left pole of the scale (e.g., inaccurate) = 0.00 and the right side of the pole = 1.00 (e.g., accurate)*.

Planned contrasts revealed that the fist above head expression significantly differed from the neutral condition on all the confidence toward shootouts scales (all *p* < 0.02; all $\eta_{p}^{2}>$ 0.39), but not on the expected penalty quality scales (accuracy *p* = 0.070; $\eta_{p}^{2}=0.20$; power *p* = 0.056; $\eta_{p}^{2}=0.22$). The chest expanded pride expression only differed on the confidence in performing to the best of their ability (*p* = 0.014; $\eta_{p}^{2}=0.34$) and confidence in scoring the next penalty measures (*p* = 0.020; $\eta_{p}^{2}=0.31$) from the neutral expression. Taken together, teammates expected to perform better in the penalty shootout when viewing a pride expression of a fellow teammate compared to a neutral expression. The hands in front of face shame expression differed from the neutral expression on all these measures (all *p* < 0.023; all $\eta_{p}^{2}>$ 0.30). The gaze down shame expression significantly differed on all these measures from the neutral condition (all $\eta_{p}^{2}>$ 0.40 for the significant measures), except for the expected penalty power (*p* = 0.114; $\eta_{p}^{2}=0.16$). All in all, teammates expected to perform worse when viewing a shame expression as compared to a neutral expression.

### Discussion

As predicted, the results of Experiment 2 on the whole revealed that teammates who observed players displaying pride anticipated feeling more pride and, in turn, expected to be more confident, in control, as well as having higher performance expectations in the shootout compared to when observing players displaying a neutral expression. In addition, shame displays caused teammates to anticipate feeling more ashamed and in turn experiencing less positive cognitions, and lower performance expectations compared to a neutral expression.

Taken together, the pattern of results of Experiment 2 is reversed compared to Experiment 1 and highlights that the social situation has to be taken into account when investigating the interpersonal effects of pride and shame expressions (Van Kleef, 2009; Moll et al., 2010).

A potential limitation of Experiment 2 (and 1) is that participants were not informed about whether the behavioral responses from the penalty kick taker followed in response of a scored or a missed penalty kick. It could be that teammates anticipated emotions, cognitions, and performance expectations were more positive (negative) after observing a pride (shame) expression because they inferred that the observed player had scored (missed) his kick, rather than being a direct effect of the observed expression.

Second, a limitation of Experiment 2 (and 1) is that both the perceived emotions as well as the anticipated emotions were assessed with several exploratory measures that have not been established in previous research on emotion expressions.

Therefore, the rationale of Experiment 3 was to address these limitations by informing observers about the outcome (always a score) and using established scales to further examine how pride and shame expressions influenced teammates' anticipated emotions during a soccer penalty shootout focusing solely on the distinct emotions: pride, happiness, and anxiety.

## Experiment 3: The effect of nonverbal pride and shame expressions on team-mates after scoring a penalty

In contrast with Experiment 2, teammates (participants) were informed about the outcome of the kick (score) when observing the behavioral responses of the penalty kick takers. Furthermore, we solely focus on how pride and shame expressions influence teammates anticipated emotions by using established scales to assess the distinct emotions: pride, happiness, and anxiety.

First, we hypothesized that pride and shame expressions could be distinguished based on biological motion information from neutral expressions on the corresponding emotion measures. Further and similar to Experiment 2 we predicted that teammates would anticipate experiencing more pride and happiness observing teammates expressing pride compared to a neutral expression. After observing displayed shame, we predicted that teammates would anticipate experiencing less pride and happiness compared to a neutral expression. In addition, we explored the effects of pride and shame expressions on anticipated anxiety.

### Methods

#### Participants

Fifteen experienced male soccer players (*M*_age_ = 22.13; *SD* = 1.25) took part in the study. They had on average 14.47 years (*SD* = 2.20) of playing experience at a competitive level. Neither age nor playing experience significantly moderated the pattern of results. Informed consent was obtained from every participant before commencing the experiment. The study was carried out in accordance with the Helsinki Declaration of 1975.

#### Materials and stimuli

For the stimuli in Experiment 3, we used three different post-performance NVB's from the point-light stimuli created in Experiment 2. These were: the first pride expression (cf. left most image of the top panel of Figure 2)—chosen because of the highest pride recognition ratings and the most beneficial effects upon teammates in Experiment 2 (see also Tracy et al., 2009; Moll et al., 2010); the neutral condition; and the first shame expression (cf. left most image of the bottom panel of Figure 2)—chosen because of being the better recognized shame expression of the two previously used (Tracy et al., 2009) and being frequently displayed after having scored a penalty kick (Moll et al., 2010,^1^). In addition, the results of Experiment 1 and 2 for the hands in front of face condition (high ratings for excited, stressed, and on edge) might indicate that this expression was not perceived as shame—typically regarded as a low intensity emotion—was not perceived as shame, but instead as despair^2^.

#### Measures

Similar to Experiment 2, participants rated the observed players as well as their feelings regarding the next penalty kick except for the following change: the response stem on the semantic differential scales was modified to adapt to the changing emotion measures from 0 (*not at all*) to 1 (*extremely*).

##### Perceived emotions of target player

Similar to Experiment 2, the first set of items provided data on the perceived emotions of the observed penalty taker (manipulation check; see Table 7).

**Table 7:** **Univariate analysis of Experiment 3 for the effects of post-performance NVB's on the perceived emotions felt by the target player**.

**Emotion**	***M*(*SD*)NVB1**	***M*(*SD*)NVB2**	***M*(*SD*)NVB3**	***df* (model, error)**	***F***	**η^2^*p***	***p***
**PERCEIVED EMOTION TARGET PLAYER**							
Pride	0.76(0.13)	0.60(0.17)	0.25(0.11)	2, 28	52.1	0.79	<0.001
Happiness	0.78(0.13)	0.52(0.13)	0.22(0.12)	2, 28	67.2	0.83	<0.001
Anxiety	0.26(0.13)	0.33(0.18)	0.49(0.21)	2, 28	7.3	0.34	0.003
Shame	0.12(0.09)	0.26(0.16)	0.57(0.23)	2, 28	32.3	0.70	<0.001

*NVB1, fists above head; NVB2, neutral; NVB3, head down*.

*The left pole of the scale (e.g., less pride) = 0.00 and the right side of the pole = 1.00 (more pride)*.

###### Pride

To increase the reliability of measuring pride compared to the 1-item in Experiment 1 and Experiment 2, pride was calculated as the mean response to the items: confident, successful, achieving, and accomplished. These 4 items are those that loaded highest on the achievement related State Authentic Pride subscale of the Pride Scale by Tracy and Robins (2007a). We only used items of the authentic pride subscale given the context (displayed pride after a score) to measure how displayed pride would be perceived by observers. The Cronbach alpha coefficient for perceived pride was good (α = 0.90).

###### Happiness

To increase the reliability of measuring happiness, happiness was calculated as the mean response to the items: cheerful, happy, joyful, and pleased. These four items stem from the happiness subscale of the Sport Emotion Questionnaire by Jones and colleagues (SEQ, Jones et al., 2005). The Cronbach alpha coefficient for perceived happiness was good (α = 0.92).

###### Anxiety

Anxiety was calculated as the mean response to the five items—uneasy, anxious, apprehensive, tense, and nervous—from the Anxiety subscale of the SEQ (Jones et al., 2005). The Cronbach alpha coefficient for perceived anxiety was good (α = 0.93).

###### Shame

Shame was assessed with the same 1-item measure (ashamed) used in Experiments 1 and 2.

###### Expected emotions

Similar to Experiment 2, the next set of items provided data with regard to how participants anticipated feeling toward taking the next penalty in line in the shootout. Participants rated their expected feelings of pride (α = 0.84), happiness (α = 0.93), and anxiety (α = 0.94) toward the next penalty with the same items used to measure the perceived emotions of the target player. The only modification was that the pride items: “successful,” “achieving,” and “accomplished” were changed into “I feel like being successful,” “I feel like achieving,” and “I feel like accomplishing.”

#### Procedure

The procedure in Experiment 3 was identical to Experiment 2 except for the following changes: (i) Every participant only viewed 12 videos in a random order. (ii) Participants were instructed that they would be observing point-light video clips of different penalty takers scoring a penalty kick in a soccer penalty shootout and that they had to assume being a teammate of the penalty kick taker and the next one in line to take a kick for their team.

### Results

#### Perceived emotions of the target player and manipulation check

The univariate analysis and descriptive statistics of the four perceived emotions felt by target players are displayed in Table 7.

Planned contrast revealed that the fist above head expression was rated significantly higher than the neutral condition on the pride scale [*F*_(1, 14)_ = 10.427, *p* = 0.006, $\eta_{p}^{2}=0.43$] and the shame expression significantly lower than the neutral condition [*F*_(1, 14)_ = 41.08, *p* = 0.001, $\eta_{p}^{2}=0.75$]. Planned contrast revealed that the fist above head expression was rated significantly higher than the neutral condition on the happiness scale [*F*_(1, 14)_ = 28.521, *p* = 0.001, $\eta_{p}^{2}=0.67$] and the shame expression significantly lower than the neutral condition [*F*_(1, 14)_ = 46.84, *p* = 0.001, $\eta_{p}^{2}=0.77$]. Planned contrast revealed that the fist above head expression did not significantly differ from the neutral condition on the anxiety scale [*F*_(1, 14)_ = 2.678, *p* = 0.124, $\eta_{p}^{2}=0.16$], but the shame expression was rated significantly higher than the neutral condition [*F*_(1, 14)_ = 5.724, *p* = 0.031, $\eta_{p}^{2}=0.29$]. Planned contrasts on the perceived shame item revealed significant differences from neutral for both the pride expression [*F*_(1, 14)_ = 12.087, *p* = 0.004, $\eta_{p}^{2}=0.46$] and the shame expression [*F*_(1, 14)_ = 24.616, *p* = 0.001, $\eta_{p}^{2}=0.64$] with the shame expression being rated higher and the pride expression lower.

#### Expected emotions toward the next penalty kick

**Figure 4:**
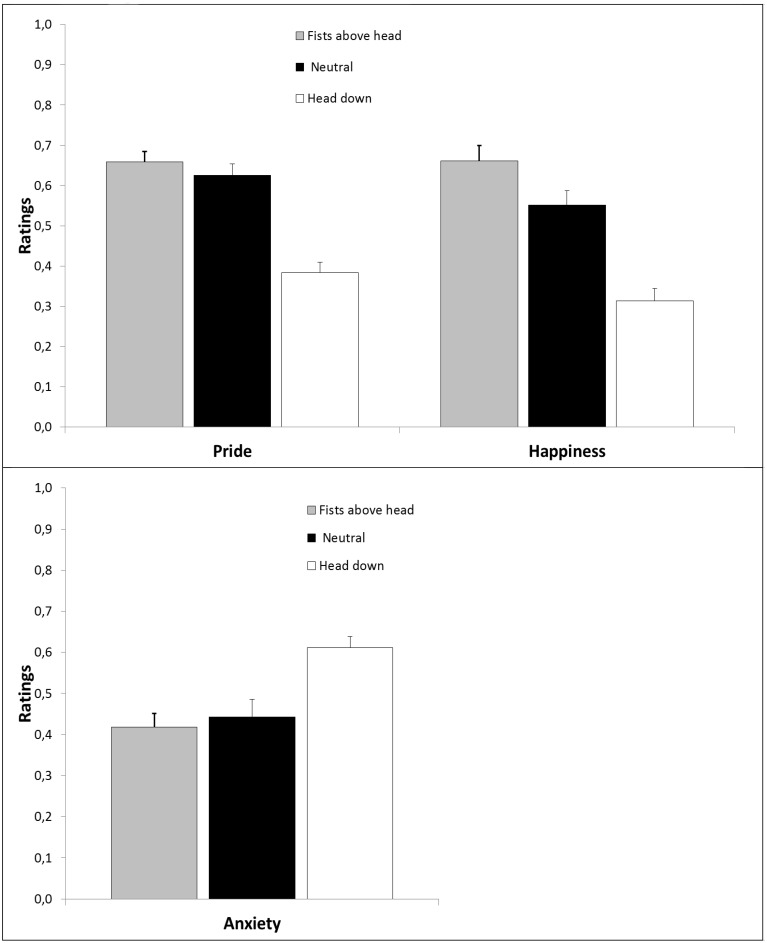
**Top:** Expected pride, happiness, and anxiety in Experiment 3 as a function of post-performance NVB; **Bottom:** Expected anxiety as a function of NVB. Error bars represent standard errors.

The univariate analysis and descriptive statistics of the three expected emotions felt toward the next penalty are displayed in Figure 4.

The One-Way ANOVA for post-performance NVB on expected pride revealed a significant effect [*F*_(2, 28)_ = 31.13, *p* < 0.001, $\eta_{p}^{2}=0.69$]. Planned contrast revealed that the fists above head expression did not significantly differ from the neutral condition on the pride scale [*F*_(1, 14)_ = 0.877, *p* = 0.365, $\eta_{p}^{2}=0.06$], but the shame expression was rated significantly lower than the neutral condition [*F*_(1, 14)_ = 37.81, *p* = 0.001, $\eta_{p}^{2}=0.73$].

The One-Way ANOVA for post-performance NVB on expected happiness revealed a significant effect [*F*_(1.45, 20.31)_ = 27.62, *p* < 0.001, $\eta_{p}^{2}=0.66$]. Planned contrast revealed that the fist above head expression was rated significantly higher than the neutral condition on the happiness scale [*F*_(1, 14)_ = 12.47, *p* = 0.003, $\eta_{p}^{2}=0.47$] and the shame expression significantly lower than the neutral condition [*F*_(1, 14)_ = 21.81, *p* = 0.001, $\eta_{p}^{2}=0.61$].

The One-Way ANOVA for post-performance NVB on perceived anxiety revealed a significant albeit weaker effect [*F*_(2, 28)_ = 9.58, *p* = 0.001, $\eta_{p}^{2}=0.41$]. Planned contrast revealed that the fist above head expression did not significantly differ from the neutral condition on the expected anxiety scale [*F*_(1, 14)_ = 0.284, *p* = 0.602, $\eta_{p}^{2}=0.02$], but the shame expression was rated significantly higher than the neutral condition [*F*_(1, 14)_ = 10.478, *p* = 0.006, $\eta_{p}^{2}=0.43$].

### Discussion

The NVBs were perceived in the predicted manner. Experiment 3 showed that teammates anticipated feeling less proud, less happy, and more anxious toward taking the next penalty kick in line after observing a post-performance expression of shame compared to a neutral post-performance expression. However, the pattern of results was not as clear cut for the pride expression. Pride expressions only lead team-mates to feel more happy compared to the neutral expression and not more proud and less anxious. Figure 4 shows that pride expression only significantly differed from shame expressions on expected feelings of pride (*p* = 0.001, $\eta_{p}^{2}=0.78$) and anxiety (*p* = 0.001, $\eta_{p}^{2}=0.57$).

Given the effects of pride and shame expressions on anxiety and happiness on taking the next penalty kick it seems likely that teammates interpreted the displayed expressions (inferential processing) to shape their emotions (at least to some extent) about their next kick in line particularly because they first rated the emotions experienced by the observed penalty kick taker. The likelihood that cognitive processing played a role in this context is further enhanced because of asking teammates to rate their emotions in relation to the next kick in line. We did exclude the possibility that the ratings were primarily influenced by the inferred outcome of the penalty observed and not the displayed NVB as observers were informed that all players scored. In this respect it is important to note that penalty takers frequently display the shame expression (gaze down, shoulder slumped) when scoring a penalty in actual game situations (Moll et al., 2010)^1^.

The rationale of Experiment 4 was to rule out the possibility that participants may have been influenced by first rating the emotions experienced by the observed penalty kick taker, and to examine the direct link between the observed emotion expressions and teammates' anticipated emotions. Therefore, teammates solely rated how they expected to feel after observing the differing NVBs in Experiment 4.

## Experiment 4: Feelings of players after observing a team-mate displaying pride or shame

The hypotheses were identical to Experiment 3.

### Method

#### Participants

Twenty four experienced male soccer players (*M*_age_ = 22.00; *SD* = 2.11) took part in the study. They had on average 14.08 years (*SD* = 2.24) of playing experience at a competitive level. Neither age nor playing experience significantly moderated the pattern of results. Informed consent was obtained from every participant before commencing the experiment. The study was carried out in accordance with the Helsinki Declaration of 1975.

#### Materials and stimuli

The materials and procedure in Experiment 4 were identical to Experiment 3, except that participants were only asked to rate how the penalty kick takers would make them feel after watching the penalty kick taker score. Participants rated their expected feelings of pride (α = 0.93), happiness (α = 0.97), and anxiety (α = 0.93) on the same measures as in Experiment 3.

### Results

#### Expected emotions

**Figure 5:**
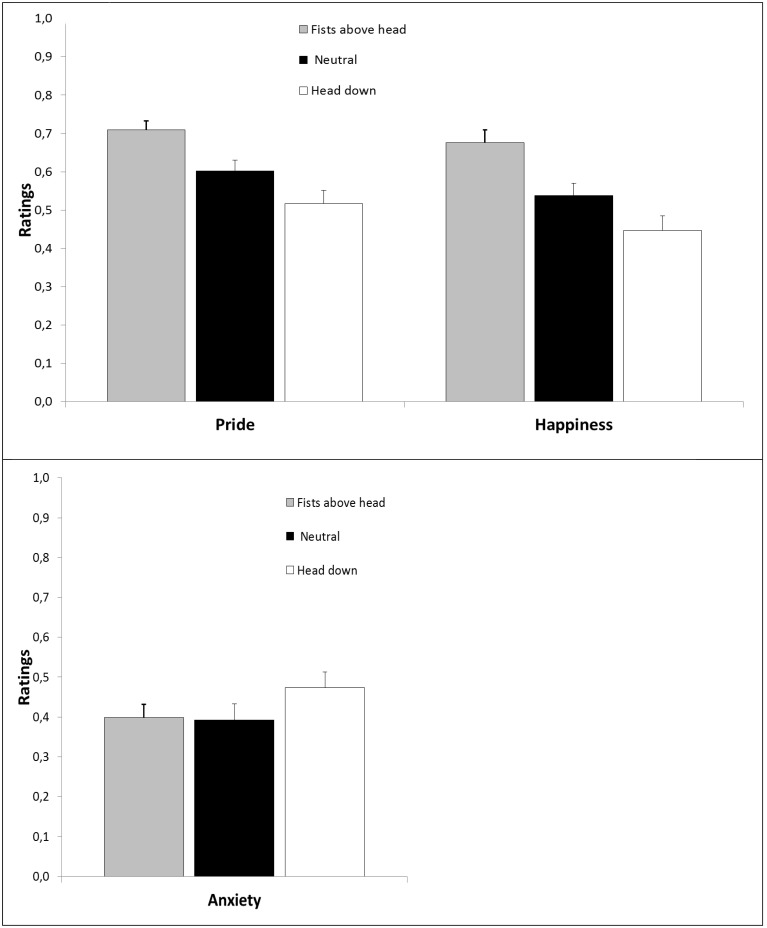
**Top:** Expected pride and happiness in Experiment 4 as a function of post-performance NVB; **Bottom:** Expected anxiety as a function of NVB. Error bars represent standard errors.

The univariate analysis and descriptive statistics of the three expected emotions felt in response to observing the penalty kick taker immediately after scoring his kick are shown in Figure 5.

The One-Way ANOVA for post-performance NVB on expected pride revealed a significant effect [*F*_(1.52, 34.95)_ = 20.06, *p* < 0.001, $\eta_{p}^{2}=0.47$]. Planned contrast revealed that the fist above head expression was rated significantly higher than the neutral condition on the pride scale [*F*_(1, 23)_ = 14.11, *p* = 0.001, $\eta_{p}^{2}=0.38$] and the shame expression significantly lower than the neutral condition [*F*_(1, 23)_ = 13.37, *p* = 0.001, $\eta_{p}^{2}=0.37$].

The One-Way ANOVA for post-performance NVB on expected happiness revealed a significant effect [*F*_(1.59, 36.47)_ = 27.14, *p* < 0.001, $\eta_{p}^{2}=0.54$]. Planned contrast revealed that the fist above head expression was again rated significantly higher from the neutral condition on the happiness scale [*F*_(1, 23)_ = 24.78, *p* = 0.001, $\eta_{p}^{2}=0.52$] and the shame expression significantly lower than the neutral condition [*F*_(1, 23)_ = 11.94, *p* = 0.002, $\eta_{p}^{2}=0.34$].

The One-Way ANOVA for post-performance NVB on perceived anxiety revealed a significant effect [*F*_(2, 46)_ = 4.24, *p* = 0.021, $\eta_{p}^{2}=0.16$]. Planned contrast revealed that the fist above head expression did not significantly differ from the neutral condition on the expected anxiety scale [*F*_(1, 23)_ = 0.033, *p* = 0.86, $\eta_{p}^{2}=0.001$], but the shame expression was rated significantly higher than the neutral condition [*F*_(1, 23)_ = 10.178, *p* = 0.004, $\eta_{p}^{2}=0.31$].

### Discussion

The results of Experiment 4 showed that teammates also anticipated feeling less proud, less happy, and more anxious after observing a post-performance expression of shame compared to a neutral post-performance expression, when not being asked to rate the emotion expression of the target player. This time, teammates also anticipated feeling significantly more pride (and happiness; a point we return to in the General Discussion) after observing a penalty taker displaying pride compared to a neutral expression. As this pattern was also evident in Experiment 3, and Experiment 4 had a higher power to detect this effect, we do not consider the findings of Experiment 3 as “evidence of absence” for an interpersonal effect of pride expressions compared to neutral expressions (see Stanley and Spence, 2014 for a detailed discussion of this).

The findings are similar to those observed in Experiments 2 and 3, but extend these findings by showing a more direct link between the observed expression and teammates' anticipated emotions suggesting that cooperative observers may have caught the emotion they observed (Van Kleef, 2009). Fitting with this direct link is that observing a pride expression as a cooperative individual resulted in higher pride (and happiness ratings) but not in lower anxiety ratings. We will return to this point in the general discussion.

## General discussion

The general aim of this study was to examine whether post-performance nonverbal expressions of pride and shame influence cooperative and competitive observers in a hypothetical soccer penalty shootout and thereby add to the understanding of the reported association between outcomes in soccer penalty shootouts and pride and shame expressions (Moll et al., 2010). Across four experiments, pride and shame expressions exerted strong effects upon observers' anticipated emotions, associated cognitions, and performance expectations, presumably because these expressions are implicitly associated with status (Pilot Study 1) and performance related attributes (Pilot Study 2). In line with Van Kleef's (2009) *EASI* model the present studies provide evidence that displays of pride and shame can exert substantial interpersonal effects upon observers that differ depending on the context.

In an initial step we demonstrated that the point-light expressions of pride and shame are implicitly associated with status and performance related attributes. These findings are important as they suggest that the results of Experiments 1–4 are not likely to be solely explained by demand effects of the experimental within-subject design. Instead, although we did not directly control for the alternative explanation of general positivity or negativity in the present IAT studies, previous research by Shariff and Tracy (2009) has rendered this unlikely. Given the similarity of the present IAT findings to the findings by Shariff and Tracy (2009), it seems more plausible that pride and shame expressions have discrete interpersonal effects on both team-mates and opponents that go beyond the simplistic notion that positive expressions are good and negative expressions are bad as they were automatically linked with status and performance. Therefore, this implicit association was likely to have been responsible for some of the variance in participant's ratings. In addition, if our findings would be solely explained by general positivity and negativity, one would have expected to find that participants in Experiments 3 and 4 would have also anticipated feeling less anxious after observing a pride expression of a team-mate, which was not the case. However, we acknowledge that further work is needed to gain a better understanding on the discrete interpersonal effects of pride and shame expressions in real-world performance environments such as sports.

In Experiment 1, observing pride expressions led participants who assumed the role of an opponent player to expect feeling less good in terms of lower levels of pride and happiness, more stressed, less confident, less in control, less focused, less comfortable, and having lower performance expectations in the shootout compared to when observing players displaying a neutral expression. Opposing results were observed for shame expressions in comparison with neutral expressions. These findings are in agreement with the EASI model and suggest that in a competitive context, pride and shame expressions cause opposing feelings and thoughts. It seems likely that opponents extracted and processed the information conveyed by the displayed expressions (inferential processing), which, in turn, influenced the way opponents felt and thought about their upcoming penalty kick. We can, however, not rule out that through an affective reaction, the expressed emotions may have led to corresponding emotions (Van Kleef, 2009).

In contrast, the findings of Experiments 2–4 revealed that teammates who observed players displaying pride anticipated feeling more pride, more happiness, and, in turn, expected to be more confident, in control, as well as having higher performance expectations in the shootout compared to when observing players displaying a neutral expression. In addition, shame displays caused teammates to anticipate feeling more ashamed and in turn experiencing less positive cognitions, and lower performance expectations compared to a neutral expression. In line with the EASI model (Van Kleef et al., 2010; Visser et al., 2013), it seems feasible that the pride and shame expressions infected team-mates in the soccer penalty shootouts and, in turn, influenced their thoughts and feelings (regarding the situation). However, the present series of studies does not provide direct evidence for this assumption. By informing participants about the outcome of the penalty (Experiments 3 and 4) and asking them directly how they would feel (Experiment 4), we excluded some sources that render inferential processing more likely. Still, there is reason to believe that in these cooperative situations, also inferential processes played a role in shaping the observers' emotions and thoughts. For example, In Experiment 3, the observed effects upon teammates may have been fueled by both affective reactions and inferential processing as the display of pride may have signaled that something good occurred—“the penalty kick taker scored easily,” and therefore, teammates felt more happy toward taking the next kick in line but not necessarily more proud and less anxious. As Experiment 4 examined the direct link between the observed emotion and teammates' anticipated emotion, the results that teammates felt more proud (and happy) but not less anxious after observing pride could indicate that observers caught the expressed emotion they observed. Still, also here, we cannot rule out that inferences predicted the felt emotions as teammates may have inferred that the display of pride signaled dominance and power causing them to feel more proud.

Hence, the present findings do not allow specifying the relative contribution of either inferential processing or emotional contagion in mediating the pattern of results in this series of experiments and in Moll et al. (2010). Most likely, both processes play an important role in influencing others in soccer penalty shootouts and future research is needed on their respective contributions in cooperative and competitive performance contexts. The findings do provide strong evidence that the nature of the situation—competitive vs. the cooperative—plays a fundamental role in shaping the interpersonal effects of pride of shame. In this respect, it seems likely that the real-world effect of pride and shame expressions in soccer penalty shootouts reported in Moll et al. (2010) was likely caused by a complex interplay of affective and inferential processes occurring when observing opponents and team-mates, and not solely by the process of emotional contagion as proposed by Moll and colleagues.

An issue that requires discussion was that the happiness ratings were similar to the pride ratings for each displayed NVB—e.g., penalty kick takers displaying pride yielded high pride ratings and equally high happiness ratings. This might suggest that the pride expression with fists above the head may also be regarded as an expression of happiness which fits with the findings of previous work (Wallbott, 1998; Coulson, 2004). Another explanation is that the point light displays used in the present experiments did not allow for the visibility of the small smile, an essential component of the prototypical pride expression (Tracy and Robins, 2007b), and therefore the pride expression yielded equally high pride and happiness ratings. Hence, the present experiments suggest that biological motion information alone does not seem to be sufficient to distinguish the distinct emotion pride from happiness, and that facial features seem necessary to disambiguate these emotions.

Martens et al. (2012) noted that displaying shame after failure has personal benefits by avoiding social rejection by the group of significant others (Gilbert, 2007). In sport teams, displaying shame may certainly appease teammates and avoid their social rejection. However, if this means that the display of shame weakens teammates and strengthens opponents, it is worth considering whether individuals should display shame after failure. To downplay the shame expression might require initial personal sacrifices (Kalokerinos et al., 2014) but if this ultimately results in winning the competitive encounter, it certainly seems worthwhile. Needless to say, it is vital for future research to focus on observers' actual emotions and behaviors such as performance within the representative contexts.

Across the four experiments, the expressions of shame seemed to have stronger effects on observers compared to the expressions of pride. These findings fit well with the pattern that the impact of “bad is stronger than good” (see for a review, Baumeister et al., 2001) suggesting that there may be asymmetries in the relative strength of negative vs. positive emotional expressions (van Kleef, 2014). Other evidence for this suggestion comes from a negotiation study by van Kleef et al. (2004) who showed that expressions of anger had a stronger impact than expressions of happiness on the counterpart's negotiation behavior. Interestingly, in our series of experiments, the asymmetrical pattern was observed in both cooperative and competitive observers. Thus also competitive observers benefited more from viewing the shame expression of opponents, than being “put off” by viewing their pride expressions.

Despite the merits of the present research, several limitations have to be acknowledged. First and foremost, it has to be noted that the present findings are derived from an artificial laboratory situation which is obviously quite different from the intense emotions experienced and expressed during actual penalty shootouts. However, the present study is in line with Moll et al. (2010) who retrospectively analyzed the influence of pride and shame expressions during actual penalty shootouts. In tandem with this field observation, the present findings can be regarded as providing converging evidence for the interpersonal effects of expressing pride and shame.

Following from the point above, the large effect sizes found across the studies, especially in Experiments 1 and 2, require discussion. In this respect, it is important to acknowledge that high levels of experimental control come at the cost of ecological validity. Therefore, a limitation of the present design was that it made sure that no other information could be integrated to inform the participant's ratings and therefore the NVB effect was most likely exaggerated compared to the actual effects of NVB in the field. Pertinent to the present results, Kahneman (2011) argues that people in general do not acknowledge that they might be missing important information in social encounters. Instead, they tend to treat the limited information available as if it where all there is to know which Kahneman explains with reference to his WYSIATI (“What you see is all there is”) rule. This argumentation is supported by the comparison of Experiments 1 and 2 with Experiments 3 and 4 as Experiments 3 and 4 revealed smaller effect sizes in which participants were aware of the outcome of the penalty kick. In addition, the sample sizes across all experiments were small and therefore it is possible that the reported effect size estimates are inflated.

## Conclusion

In conclusion, the present research adds to the growing body of literature on nonverbal behavior in sports (Furley and Schweizer, 2014a) and its potential influence on observers (Furley and Schweizer, 2014b). Specifically, the series of studies highlights the potential interpersonal influence of the nonverbal expressions of pride and shame in competitive social situations and importantly that these depend on the social context, i.e., depending on whether these are displayed by cooperative or competitive others. Further, the results suggest that athletes are well advised to display pride after success in high-stakes sport situations, but importantly should also avoid showing shame as these expressions will influence observers and in turn might affect the final outcome of their endeavors.

## Author contributions

PF and TM developed the study concept, and both authors contributed to the design. PF and TM collected the data and analyzed it in collaboration with DM. PF and TM wrote the first draft of the manuscript, and DM helped edit and revise it. All authors approved the final, submitted version of the manuscript.

### Conflict of interest statement

The authors declare that the research was conducted in the absence of any commercial or financial relationships that could be construed as a potential conflict of interest.

## Footnotes

^1^Moll et al. (2010) showed that in penalty shootouts occurring in World Cups and European Championships, 109 of the 151 penalty kick takers (72%) gazed down after scoring when the standing was equal.

^2^We thank a reviewer for this suggestion.
